# CTCs Detection and Whole-exome Sequencing Might Be Used to Differentiate Benign and Malignant Pulmonary Nodules

**DOI:** 10.3779/j.issn.1009-3419.2023.106.12

**Published:** 2023-06-20

**Authors:** Changdan XU, Xiaohong XU, Weipeng SHAO, Hongliang SUN, Xiaohong LIU, Hongxiang FENG, Xianbo ZUO, Jingyang GAO, Guohui WANG, Xiongtao YANG, Runchuan GU, Shutong GE, Shijie WANG, Liwei GAO, Guangying ZHU

**Affiliations:** ^1^Department of Radiation Oncology, Peking University China-Japan Friendship School of Clinical Medicine, Beijing 100029, China; ^1^Department of Radiation Oncology, Peking University China-Japan Friendship School of Clinical Medicine, Beijing 100029, China; ^2^Department of Radiation Oncology, Zhongshan Hospital, Fudan University, Shanghai 200032, China; ^2^Department of Radiation Oncology, Zhongshan Hospital, Fudan University, Shanghai 200032, China; ^3^Department of Thoracic Surgery; ^3^Department of Thoracic Surgery; ^4^Department of Radiology, China-Japan Friendship Hospital, Beijing 100029, China; ^4^Department of Radiology, China-Japan Friendship Hospital, Beijing 100029, China; ^5^Department of Information Science and Technology, Beijing University of Chemical Technology, Beijing 100029, China; ^5^Department of Information Science and Technology, Beijing University of Chemical Technology, Beijing 100029, China; ^6^Department of Dermatology; ^6^Department of Dermatology; ^7^Department of Pharmacy, China-Japan Friendship Hospital, Beijing 100029, China; ^7^Department of Pharmacy, China-Japan Friendship Hospital, Beijing 100029, China; ^8^Department of Radiation Oncology, Tianjin First Central Hospital, Tianjin 300070, China; ^8^Department of Radiation Oncology, Tianjin First Central Hospital, Tianjin 300070, China; ^9^Department of Oncology, Beijing Changping District Hospital, Beijing 102200, China; ^9^Department of Oncology, Beijing Changping District Hospital, Beijing 102200, China; ^10^Faculty of Medicine Lund University, Lund SE-22100, Sweden; ^10^Faculty of Medicine Lund University, Lund SE-22100, Sweden; ^11^Department of Radiation Oncology, China-Japan Friendship Hospital, Chinese Academy of Medical Sciences & Peking Union Medical College, Beijing 100029, China; ^11^Department of Radiation Oncology, China-Japan Friendship Hospital, Chinese Academy of Medical Sciences & Peking Union Medical College, Beijing 100029, China; ^12^Department of Radiation Oncology, Center of Respiratory Medicine, China-Japan Friendship Hospital, Beijing 100029, China; ^12^Department of Radiation Oncology, Center of Respiratory Medicine, China-Japan Friendship Hospital, Beijing 100029, China

**Keywords:** Chest computed tomography, Circulating tumour cells, Lung nodule, TP53

## Abstract

**Background and objective:**

Low-density computed tomography (LDCT) improved early lung cancer diagnosis but introduces an excess of false-positive pulmonary nodules data. Hence, accurate diagnosis of early-stage lung cancer remains challenging. The purpose of the study was to assess the feasibility of using circulating tumour cells (CTCs) to differentiate malignant from benign pulmonary nodules.

**Materials and methods:**

122 patients with suspected malignant pulmonary nodules detected on chest CT in preparation for surgery were prospectively recruited. Peripheral blood samples were collected before surgery, and CTCs were identified upon isolation by size of epithelial tumour cells and morphological analysis. Laser capture microdissection, MALBAC amplification, and whole-exome sequencing were performed on 8 samples. The diagnostic efficacy of CTCs counting, and the genomic variation profile of benign and malignant CTCs samples were analysed.

**Results:**

Using 2.5 cells/5 mL as the cut-off value, the area under the receiver operating characteristic curve was of 0.651 (95% confidence interval: 0.538-0.764), with a sensitivity and specificity of 0.526 and 0.800, respectively, and positive and negative predictive values of 91.1% and 30.3%, respectively. Distinct sequence variations differences in DNA damage repair-related and driver genes were observed in benign and malignant samples. TP53 mutations were identified in CTCs of four malignant cases; in particular, g.7578115T>C, g.7578645C>T, and g.7579472G>C were exclusively detected in all four malignant samples.

**Conclusion:**

CTCs play an ancillary role in the diagnosis of pulmonary nodules. TP53 mutations in CTCs might be used to identify benign and malignant pulmonary nodules.

## Introduction

Lung cancer is one of the major cause of cancer-related deaths worldwide and ranks first in global mortality among both men and women^[1]^. In China, the morbidity of lung cancer ranks first and second place among men and women, respectively, and both in the first place in mortality^[2]^. In recent years, the prognosis of lung cancer patients has greatly improved due to the development of therapeutic facilities; however, there is a substantial difference between the prognosis of patients with early- and advanced-stage lung cancer, with a 5-year survival rate of 85% for stage IA and only 6% for stage IV^[3]^. 5-year relative survival rates for lung cancer are 6%, 33% and 60% for distant metastases, regional disease and localised disease^[1]^, respectively. Hence, early diagnosis has a crucial impact on lung cancer prognosis.

In the U.S. National Lung Screening Trial (NLST), low-density computed tomography (LDCT) was reported to reduce lung cancer mortality by 20% compared to X-rays^[4]^. As LDCT has become the main method for lung screening, an increasing number of pulmonary nodules are being detected. However, lung nodules can be either benign or malignant, thus LDCT screening is highly prone to misdiagnosis or under diagnosis lung cancer. The false positive rate in the NLST was 96.4%, which was similar to that in a Chinese lung cancer screening trial at West China Hospital (97.9%). Of the malignant cases enrolled in this Chinese study, 9.2% met the USPSTF screening criteria and 24.4% met the Chinese lung cancer screening guidelines, with missed rates of up to 90.8% and 75.6%, respectively^[4,5]^. The diagnosis of lung cancer currently relies on chest CT, positron emission tomography/CT (PET/CT), and puncture biopsy, which carries the risk of exposure to radiation and invasiveness; therefore, achieving accurate diagnosis of lung nodules between 0.5 cm and 3 cm in diameter remains challenging.

Liquid biopsy opens up a new gate for tumour diagnosis and monitoring owing to its high accuracy, good compliance, easy accessibility and repeatability. Biomarkers for liquid biopsy include circulating tumour cells (CTCs), circulating tumour DNA (ctDNA), and vesicular exosomes containing nucleic acids, proteins, and lipids secreted by tumour cells^[6]^. CTCs are tumour cells that are spontaneously or passively shedding from primary tumour lesions and enter circulation. As the “seeds” of tumour metastasis, CTCs carry a lot of messages related to tumorigenesis, growth, metastasis, and drug resistance, and have been demonstrated to be an independent prognostic factor for cancer patients^[7⇓-9]^. The latest evidence suggests that CTCs dissemination is a biological behaviour that occurs in the early stages of tumour proliferation^[10]^. Research on CTCs based on single-cell sequencing assist new biomarker discovery and reveal the metastatic mechanisms of tumour cells at the molecular level, allowing for more accurate diagnosis, personalized treatment, and improved survival of lung cancer patients^[11]^.

In this study, 122 patients diagnosed with pulmonary nodules by CT were enrolled. A modified isolation by size of epithelial tumour cells (ISET) method^[12]^ was used to detect CTCs, and CTCs whole-exome sequencing was performed on eight samples. We analysed the efficacy of CTCs counts for pulmonary nodule diagnosis and identified biomarkers to distinguish benign and malignant pulmonary nodules.

## Materials and methods

### Patients enrolment and sample collection

Patients with single or multiple pulmonary nodules (≤3 cm) suspected to be malignant on chest CT from January 2019 to November 2022 who were recommended for surgery in the Department of Thoracic Surgery at China-Japan Friendship Hospital were enrolled. CTCs testing was performed in intravenous blood samples. All patients were categorized by two radiologists according to the Lung Imaging Reporting and Data System Version 1.0 (Lung-RADS) published by the American College of Radiology^[13]^. The pathological information of all samples was determined based on the analysis of surgically resected tissue samples according to the 2015 World Health Organization Histological Classification of Lung Cancer. This study was performed comply with the principles of the Declaration of Helsinki. Approval was granted by the Ethics Committee of the China-Japan Friendship Hospital and all participants provided written informed consent.

Patients were included in the study according to the following criteria: (1) patients with lung nodules diagnosed by chest CT to be considered Lung-RADS category 2, 3 or 4; (2) Radiographically evaluable lesions with a maximum diameter ≤30 mm; (3) age ≥18 years, should have the independent civil capacity and sign the informed consent for clinical research; (4) Eastern Cooperative Oncology Group (ECOG) score 0-1, life expectancy of no less than 2 months; (5) organ function level of participants required: 1.5×10^9^/L ≤ absolute neutrophil count ≤15×10^9^/L, platelets ≥100×10^9^/L, haemoglobin ≥9.0 g/dL, prothrombin time ≥11 s; serum bilirubin ≤1.5 upper limit of normal (ULN), aspartate aminotransferase (AST) and alanine aminotransferase (ALT) ≤2.5 ULN, and serum creatinine ≤1.5 ULN. Patients were excluded from the study if: (1) had any tumour history, severe cardiovascular disease [myocardial infarction within 6 months or admission to the intensive or coronary care units for cardiovascular disease or cardiac function impairment (ejection fraction ≤50%)]; (2) had severe vascular disease, tuberculosis, massive acute inflammation (such as acute bronchitis, vasculitis) or chronic infection in the active phase within 3 months; (3) had received treatment or withdrew from a previous study; (4) participated in other drug trials within 3 months prior to enrolment; (5) were pregnant or lactating; or (6) had other conditions are considered inappropriate by the investigator.

### CTCs detection

**Fig 1:**
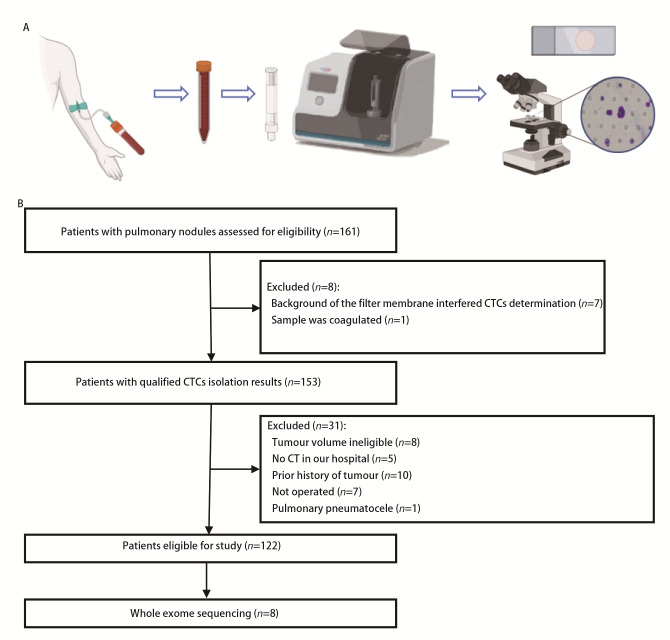
Research flowchart. A: CTCs isolation and identification approach. B: Framework of the study. Patients (n=161) consented for blood sampling, 39 patients were excluded due to technical reasons that could affect CTCs analysis results and those who did not match the inclusion criteria. A total of 122 patients were included in the final analyses, of which samples from 8 patients were sequenced. CTCs: circulating tumour cells; CT: computed tomography.

CTCs testing was conducted within a week before surgery. Whole blood samples were collected using EDTA anticoagulant blood collection tubes; 2 mL from the first tube was discarded and 5 mL from the second tube was separated within 2 h. CTCs were isolated from the whole blood samples using the CTCBIOPSY Abnormal Cell Separator (Wuhan Youzhiyou Medical Technology, Wuhan, China)^[12]^ and were identified using Wright’s stain. The isolated cells were examined by a pathologist using bright-field microscopy to determine the presence of individual cells (CTCs) and cell clusters (CTMs) with morphological characteristics of tumour cells and counting numbers (Figure 1A).

The isolated cells were defined as CTCs if they meet four or more of the following criteria (CTCs were diagnosed if there are large nucleoli or abnormal nuclear divisions, along with two of the other diagnostic criteria)^[14⇓-16]^: (1) abnormal karyotype; (2) nucleoplasma ratio >0.8; (3) cell diameter >15 μm; (4) deeply stained and unevenly coloured nuclei; (5) thickened nuclear membrane with depressions or folds, resulting in an irregular nuclear membrane; and (6) large nucleoli.

### Laser capture microdissection

Single-cell laser capture microdissection of CTCs was conducted using a LMD7000 instrument (Leica, Wetzlar, Germany). The CTCs to be captured were examined under a microscope, and an ultraviolet laser was used to automatically scan and cut the desired single-cell edge. The selected cells were then isolated and dropped into the collection tube. CTCs from the same patient were collected in the same tube for subsequent whole-genome amplification (WGA). The leukocyte microdissection method was the same as that used for the CTCs.

### WGA

WGA of the CTCs and white blood cells from each patient was performed at the oligo-cell level using MALBAC Single Cell Whole Genome Amplification Kit (Yikon Genomics, Shanghai, China), according to the manufacturer’s instructions. WGA efficacy was evaluated by 1% agarose gel electrophoresis, and amplification products 300 bp-2,000 bp in size being obtained. DNA concentration was determined using a Qubit 2.0 fluorometer (Life Technologies, Waltham, MA, USA). Samples with DNA concentration ≥5 ng/μL and a total DNA amount of ≥0.1 μg were qualified for further analysis.

### DNA library preparation, exon capture and next-generation sequencing

Genomic DNA was randomly broken into fragments of 180 bp-280 bp using Covaris (Woburn, MA, USA), end-repaired and A-tailed, and an adaptor was ligated at each side of the fragment to prepare the DNA library. Then, the library was polled, hybridized with biotin-labelled probes, captured using magnetic beads, and amplified by polymerase chain reaction (PCR) for quality control. The Qubit 2.0 was used to check preliminary quantification, followed by the detection of the insert size of the library using 2100 Bioanalyzer system (Agilent, Santa Clara, CA, USA). The concentration of the library was accurately quantified by quantitative PCR (3 nmol/L) and the sequences that passed the examination were sequenced using MGISEQ-T7 (pair end, 150 bp; MGI, Shenzhen, China).

### Bioinformatics analysis

FASTP^[17]^ was used to remove adaptors and low-quality bases for quality control. Cleaned reads were then aligned to the human genome assembly 19 (GRCh37) using Burrows-Wheeler Aligner (BWA)^[18]^ and the results were stored in BAM format. Bamdst (https://github.com/shiquan/bamdst) was used to conduct basic statistics on the obtained files according to the location of the target region. Mutect2 was used to detect single nucleotide variations (SNVs)/insertion-deletions in the GATK bundle (https://gatk.broadinstitute.org/hc/en-us). All variants were annotated using ANNOVAR^[19]^, which were then filtered to remove false-positive data as follows: (1) reads with alignment quality of less than 20 were excluded; (2) reads with a secondary alignment were excluded; (3) number of bases on both sides of the mutant position ≥20, otherwise the read was rejected; (4) no mismatches between the 20 bases on either side of the mutant position, otherwise the reads were rejected. Variants that satisfied these requirements were regarded as ‘PASS’ and only variants with at least 20 reads were considered for further analysis. To generate a high-confidence set of variant calls from CTCs, the following filters were applied: (1) variants with allele frequency (VAF) <0.2 were removed; (2) variants in 1000Genomes, Esp6500, inhouse, and EXAC >0.01 according to the annotation of ANNOVAR were removed; (3) if any variant was called in the white blood cell controls, that variant was filtered out; and (4) removal of synonymous mutations. Two-by-two comparisons between the sequenced samples were performed based on the mutant locus.

### Statistical analysis

Statistical analysis was performed using SPSS 26.0 (IBM Corp., Armonk, NY, USA). χ^2^ test was used for statistical analysis of the numerical data; Mann-Whitney U test for measurement data; and the receiver operating characteristic (ROC) curve was applied to the evaluation of the diagnostic efficiency of CTCs counts on pulmonary nodules. Binary logistic regression was used to analyse factors affecting lung cancer. RevMa 5.4 (Cochrane, London, U.K.) was used to perform the meta-analysis. KOBAS 2.0^[20]^ was used to conduct the KEGG analysis, with the Fisher’s exact test being performed for statistical analysis, and the Benjamin-Hochberg method being used to correct the P-value. Statistically significant difference was set P<0.05.

## Results

### The clinical and pathological characteristics of the patients

Between January 2019 and November 2022, a total of 122 patients with median age of 58 years (22 years-83 years) and suspected malignant pulmonary nodules (≤3 cm) found on chest CT were enrolled in the study (Figure 1B), among whom 49 were male. Postoperative pathology proved that these tumours were pathologically malignant in 97 patients (79.5%). Smoking history was recorded in 114 patients, of whom 29 (25.4%) had a previous smoking history. Radiographic data, which was available for 120 of the patients, revealed solid and subsolid nodules in 32 (26.7%) and 88 (73.3%) patients, respectively. The nodules were classified as Lung-RADS category 2, 3 and 4 in 22 (18.3%), 20 (16.7%), and 78 (65.0%) patients (Table 1).

**Tab 1:** Clinical pathological characteristics of the enrolled patients

Characteristic	Benign disease	Malignant disease	P-value
Gender, n (%)	n=25	n=97	0.504
Female	13 (52.0)	60 (61.9)	
Male	12 (48.0)	37 (38.1)	
Age, yr, median (range)	55 (22, 70)	59 (30, 83)	0.148
Pathology, n (%)	n=25	n=97	<0.001
Hamartoma	2 (8.0)	0 (0)	
Inflammation	21 (84.0)	0 (0)	
Tuberculosis	1 (4.0)	0 (0)	
AAH	1 (4.0)	0 (0)	
AIS	0 (0)	10 (10.3)	
MIA	0 (0)	28 (28.9)	
IAC	0 (0)	49 (50.5)	
Other tumour	0 (0)	8 (8.3)	
SCC	0 (0)	1 (1.0)	
SCLC	0 (0)	1 (1.0)	
Nodule diameter, n (%)	n=24	n=96	0.202
<6 mm	1 (4.2)	1 (1.0)	
≥6 mm to <8 mm	6 (25.0)	12 (12.5)	
≥8 mm to <15 mm	9 (37.5)	39 (40.6)	
≥15 mm	8 (33.3)	44 (45.8)	
Nature of nodule, n (%)	n=24	n=96	<0.001
Solid	14 (58.3)	18 (18.8)	
Subsolid	10 (41.7)	78 (81.3)	
Lung-RADS category, n (%)	n=24	n=96	0.030
2	7 (29.2)	15 (15.6)	
3	5 (20.8)	15 (15.6)	
4A	3 (12.5)	2 (2.1)	
4B	1 (4.2)	18 (18.8)	
4X	8 (33.3)	46 (47.9)	
Smoking status, n (%)	n=22	n=92	0.622
Non-smoker	15 (17.6)	70 (82.4)	
Smoker	7 (24.1)	22 (75.9)	

AAH: atypical adenomatous hyperplasia; AIS: adenocarcinoma in situ; IAC: invasive adenocarcinoma; Lung-RADS: Lung Imaging Reporting and Data System; MIA: minimally invasive adenocarcinoma; SCC: squamous cell carcinoma; SCLC: small cell lung cancer.

Whole-exome sequencing was performed on CTCs samples from 8 of the patients, including 6 cases of malignant disease. P1 was an external diagnosis of pulmonary malignancy with no in-house pathological examination, while the postoperative pathology was preserved in the remaining seven patients.

### Efficacy of CTCs count for pulmonary nodule classification

**Fig 2:**
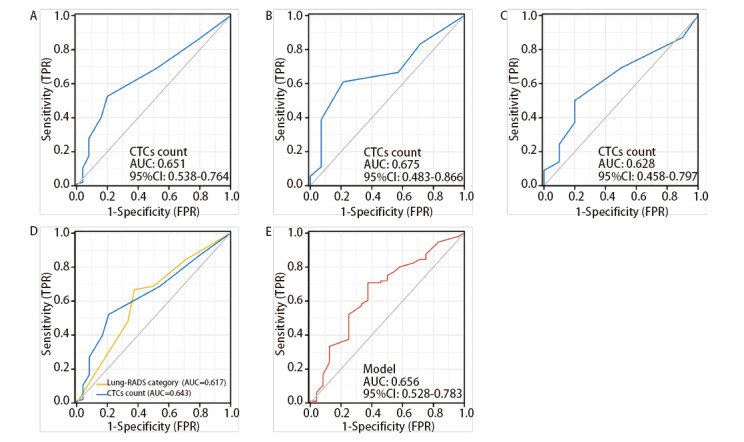
Diagnostic efficacy of CTCs counts. A-C: ROC curves based on CTCs counts in all patients (A), solid nodules (B), subsolid nodules (C); D: Comparison of ROC curves for CTCs and Lung-RADS category; E: ROC curve for the combined CTCs and Lung-RADS. ROC: Receiver operating characteristic; AUC: area under the curve; Lung-RADS: Lung Imaging Reporting and Data System; CI: confidence interval.

The CTCs count was not normally distributed among benign and malignant patients (P<0.001). The CTCs detection rate was similar between patients with benign and malignant disease (80.0% vs 86.6%, respectively; P=0.526); nevertheless, the CTCs counts differed significantly between the groups (P=0.019). Using the cut-off value of 2.5 cells/5 mL for CTCs positivity determined by the Youden index, the ROC curve for CTCs counts achieved a sensitivity of 0.526, specificity of 0.800, and area under the curve (AUC) value of 0.651 [95% confidence interval (CI): 0.538-0.764], with positive and negative predictive values of 91.1% and 30.3%, respectively (Figure 2A). The pathological malignancy rates were 91.1% and 69.7% in the CTCs-positive and CTCs-negative groups, respectively (P=0.004).

For solid nodules, the AUC was 0.675 (95%CI: 0.483-0.866), surpassing 0.628 (95%CI: 0.458-0.797) for subsolid nodules; however, the observed AUC difference was not statistically significant (P=0.716) (Figure 2B and 2C), which may be related to the limited number of solid nodule cases among the patients enrolled in the study.

The diagnostic efficacies of the CTCs count and Lung-RADS category for pulmonary nodules were similar, with AUCs of 0.643 (95%CI: 0.527-0.759) and 0.617 (95%CI: 0.490-0.743), respectively (P= 0.726) (Figure 2D). The AUC for CTCs combined with Lung-RADS was 0.656 (95%CI: 0.528-0.783) (Figure 2E).

Analysis of factors influencing the determination of lung nodules showed that among all statistical factors, CTCs and the nature of the nodules were statistically significant, indicating that CTCs positivity and subsolid nodules were independent factors influencing lung cancer development (Table 2).

**Tab 2:** Logistic regression analysis of factors influencing lung cancer incidence among patients with pulmonary nodules

Characteristics	Odds ratio (95%CI)	P-value
Nature of nodule		
Solid	1	
Subsolid	18.965 (3.456-104.089)	0.001
CTCs		
Negative	1	
Positive	3.826 (1.105-13.253)	0.034
Lung-RADS category		
2	1	
3	0.802 (0.177-3.638)	0.775
4A	1.659 (0.095-29.09)	0.729
4B	7.648 (0.617-94.856)	0.113
4X	8.141 (1.219-54.384)	0.030

### Genomic mutation characteristics of CTCs in benign and malignant pulmonary nodules

**Fig 3:**
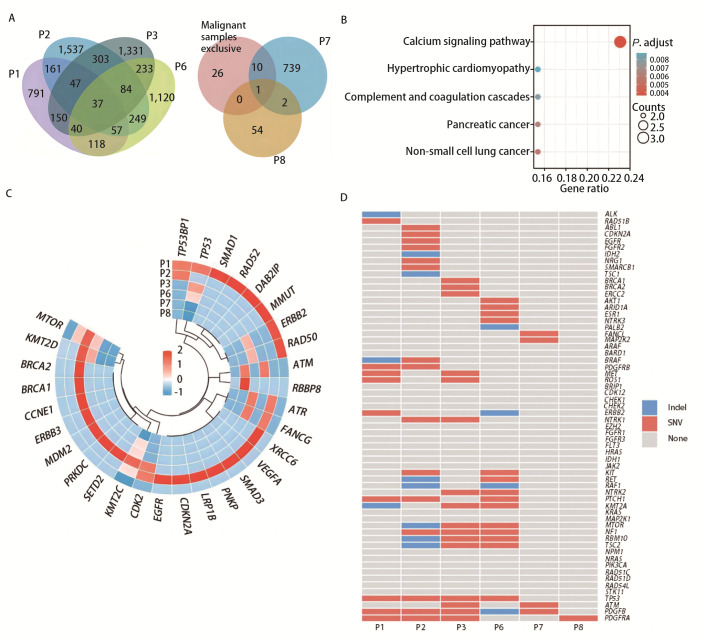
Genomic characteristics of the sequenced CTCs samples. A: Venn diagram of the number of mutated genes in all samples; B: KEGG analysis of 26 mutated genes shared in malignant samples; C: Heatmap of DNA damage repair genes and cell cycle-associated genes in 6 CTCs samples; D: Heatmap of common driver genes identified in 6 CTCs samples. KEGG: Kyoto Encyclopedia of Genes and Genomes; SNV: single nucleotide variant.

ROC analysis demonstrated that CTCs counts aid on the diagnosis of pulmonary nodules. To explore their impact on the diagnostic accuracy at the molecular level, we conducted an experimental study of whole-exome sequencing of CTCs collected from 8 patients with pulmonary nodules (6 with malignant disease and 2 with benign disease). The somatic mutation profile showed that, except for two samples (P4 and P5), which were excluded because of low sequencing coverage, 37 mutated genes were shared among CTCs from patients with malignant disease, of which 26 were not detected in CTCs samples from benign patients (Figure 3A). Among the identified potentially pathological mutated genes was TP53, which is a known oncogene that is involved in relevant pathways^[21]^, namely the cell cycle and p53 pathway. KEGG analysis of these 26 genes showed that the top significantly enriched pathway was the calcium signalling pathway (RYR1, RYR3 and PHKG1) (P<0.05, P_corrected_<0.05) (Figure 3B).

Genomic instability is a major contributor to cancer development; thus, we explored the mutation profiles of DNA damage repair and cell-cycle pathway genes associated with cancer development. The number of variants in DNA damage repair genes was generally higher in the malignant disease samples, whereas only three genes were mutated in the benign sample (P7: RBBP8, ATM, and KMT2C) (Figure 3C). Although a small number of ‘circulating abnormal cells’ were also released into the peripheral blood in patients with benign disease, the malignancy severity was significantly lower.

### Variants of driver genes in CTCs

Driver gene mutations play key roles in cancer development. We investigated frequent lung cancer driver genes reported in previous studies of early-stage lung cancer^[22,23]^ and among the OncoKB database^[24]^ level 1-3 pan-cancer driver genes to explore the feasibility of testing driver mutations in CTCs for classifying pulmonary nodules.

In malignant disease CTCs, TP53 variations were present in all four malignant samples (P1-P3 and P6) and were not found in the benign disease samples (P7 and P8). Of the remaining 62 common driver genes, 16 mutant genes were present in more than two samples of malignant CTCs and none were found in the two benign samples. In addition, a small number of driver mutations were identified in CTCs samples from patients with benign disease and no RAS mutations were identified in any of the samples (Figure 3D).

### Role of mutated TP53 in the identification of pulmonary nodules

Inactivation of p53 promotes genomic instability and is generally recognized as being associated with tumorigenesis. From the above results, we found that mutated TP53 could be used to initially differentiate malignant from benign pulmonary nodules. To further analyse the specific role of TP53 in the diagnosis of lung nodules, we compared each sample according to the mutated loci; P4 and P5 were excluded due to the low sequencing coverage. Three TP53 mutations were identified exclusively in all four malignant samples: g.7578115T>C g.7578645C>T, and g.7579472G>C (GRCh37.p13 chr 17). The mutation types were intronic, 5′ untranslated region, and exonic SNVs. In addition, loss of heterozygosity (VAF approximate 1) at the TP53 loci was observed in three of the samples (Table 3).

**Tab 3:** Information of TP53 mutant loci in malignant samples

Patient	Mutant position	Base change			Alteration	Reference	Depth	VAF	Mutation type	Effect
		Reference	Alteration							
P1	7578115	T	C		84	0	84	1	Intronic SNV	Unknown
P2					16	8	24	0.667		
P3					179	0	179	1		
P6					48	1	49	0.980		
P1	7578645	C	T		2,028	0	2,028	1	5′-UTR SNV	Unknown
P2					44	68	112	0.393		
P3					305	0	305	1		
P6					192	1	193	0.995		
P1	7579472	G	C		577	1,190	1,767	0.327	Exonic SNV	Non-synonymous
P2					66	59	125	0.528		
P3					350	1	351	0.997		
P6					74	0	74	1		

UTR: untranslated region; VAF: variant allele frequency.

**Fig 4:**
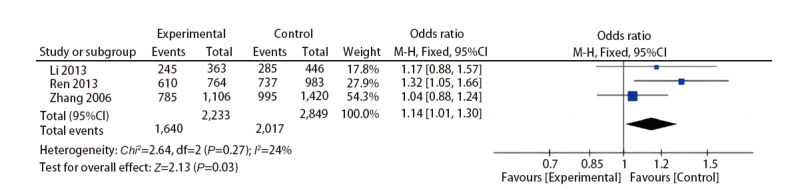
Meta-analysis of the risk represented by the TP53 rs1042522 variant

A search of the dbSNP database (https://www.ncbi.nlm.nih.gov/snp/) revealed that rs1042522 is one of the most common SNPs of TP53. A subsequent meta-analysis of case‒control studies for this locus^[25⇓-27]^ showed that a G>C variation in either allele at this site (CC/GC) increased the risk of lung cancer, with an odds ratio of 1.14 (95%CI: 1.01-1.30) (Figure 4). This phenomenon was not only observed in pathological tissues, but our study also showed that CTCs from patients with early-stage lung cancer carry risk locus mutations, whereas ‘circulating abnormal cells’ from patients with benign disease do not.

## Discussion

To the best of our knowledge, our study is the first to explore the difference between benign and malignant lung nodules by CTCs oligo-cell sequencing. Early diagnosis of lung cancer has been the most effective way to reduce cancer mortality to date. Liquid biopsy represents one of the most attractive tools for cancer diagnosis owing its high accuracy and compliance. CTCs are derived from the primary tumour and have more integrated genomic information than ctDNA. Therefore, CTCs detection with high sensitivity and specificity can benefit people at risk and those with early-stage lung cancer^[28]^.

Cell morphological alterations are an important basis for differentiating benign and malignant tumours. During the early stage, lung cancer releases a small amount of CTCs owing its overall small tumour volume; thus, the current commonly used CellSearch system can easily fail to detect CTCs^[29]^. Therefore, in the present study, we combined ISET with morphological identification to detect CTCs and identify lung cancer precursors^[30]^. A previous study^[31]^ reported that a portion of circulating non-haematological cells, lymphocytes, and monocytes are within the cells captured by ISET, which may become background cells in the test results. In our study, a cut-off value of 2.5 cells/5 mL was determined to be used to differentiate CTCs-positive from CTCs-negative samples, thus avoiding this problem to the utmost.

Our study revealed that CTCs can auxiliary differentiate precursors from benign disease and is ancillary to clinical diagnosis of pulmonary nodules, which agrees with the findings of previous studies^[32⇓-34]^. CTCs showed no inferior diagnostic potential to that of the Lung-RADS classification. The ROC curve indicated that CTCs were superior for the diagnosis of solid nodules compared with subsolid nodules, but the difference was not statistically significant. Xing et al.^[35]^ discovered from single-cell RNA sequencing of subsolid nodules samples that lung adenocarcinoma showing subsolid nodules on CT imaging exhibited a more indolent biological behaviour and milder immune stress compared with lung cancer cases with solid nodules. This may explain the slightly less effective diagnosis of subsolid nodules by CTCs (owing their weaker proliferative and metastatic nature). Additionally, no significant differences in the mutational signature between solid and subsolid nodules were found, probably due to the small number of cases (n=8). Further studies with an expanded sample size are still needed.

The mutation profiles of cell cycle and DNA damage repair-correlated genes in benign and malignant lung nodules in this study showed a clear disparity. Although a small number of driver mutations were detected in the benign samples, the degree of malignancy needs to be carefully evaluated. TP53 mutations were present in all four malignant CTCs samples and were not detected in CTCs from patients with benign disease; however, this percentage was 30% in targeted ctDNA sequencing^[36]^.

The tumour suppressor gene TP53 is located in chromosome 17p13 and plays critical roles in the regulation of cell cycle checkpoints, DNA damage repair, and apoptosis^[37]^. Baslan et al.^[38]^ reported that sporadic loss and accumulation of deletions in p53 are oncogenic events. Although TP53 mutations have been extensively studied in lung cancer, the role of TP53 polymorphisms in the diagnosis of lung nodules and the impact of co-mutations with other driver genes on cancer pathogenesis have not been elucidated. Our study identified three TP53 SNPs present only in malignant CTCs samples, all of which were associated with lung cancer susceptibility^[39⇓⇓-42]^.

Our study demonstrates the viability of CTCs in assisting diagnosis of benign and malignant pulmonary nodules. In this exploratory study, we identified three TP53 variants exclusively in CTCs of patients with malignant disease. The mutation pattern of TP53 in CTCs will be further investigated by expanding the sample size in the future. In addition, lung cancer driver gene alterations have been found in most CTCs samples of malignant diseases. Therefore, CTCs detection combined with driver gene panel testing holds promise for improving the accuracy and convenience of future early-stage lung cancer diagnosis.

There are also limitations to this study, such as the limited number of enrolled cases, non-complete CTCs genome after amplification, low sequencing coverage^[43]^, and the invalidated concordance of driver gene alterations in CTCs with pathological tissues. Herein, ‘circulating abnormal cells’ are detected in patients with benign disease possibly because: (1) large cells in the blood, such as lymphocytes, monocytes, and shedding of vascular endothelial cells may lead to false positive results^[44]^, and (2) these patients may have undetected tumour lesions that release CTCs and require repeated testing and follow-ups. With the development of artificial intelligence technology, the combination of such novel diagnostic technology with CT and CTCs data may help achieve better diagnostic results.

In conclusion, our study shows that CTCs counts combined with TP53 mutations might help the clinical diagnosis of lung nodules.

## Acknowledgement

We would like to thank the YZY MED company for providing technical support and assistance.

## Conflicts interests

The authors have no conflict interests to disclose.
